# Exploratory Bioinformatics Study of lncRNAs in Alzheimer's Disease mRNA Sequences with Application to Drug Development

**DOI:** 10.1155/2013/579136

**Published:** 2013-04-11

**Authors:** T. Holden, A. Nguyen, E. Lin, E. Cheung, S. Dehipawala, J. Ye, G. Tremberger, D. Lieberman, T. Cheung

**Affiliations:** ^1^Queensborough Community College of CUNY, 222-05 56th Avenue Bayside, NY 11364, USA; ^2^Albert Einstein College of Medicine, Department of Medicine, 1300 Morris Park Avenue, Bronx, NY 10461, USA

## Abstract

Long noncoding RNA (lncRNA) within mRNA sequences of Alzheimer's disease genes, namely, APP, APOE, PSEN1, and PSEN2, has been analyzed using fractal dimension (FD) computation and correlation analysis. We examined lncRNA by comparing mRNA FD to corresponding coding DNA sequences (CDSs) FD. APP, APOE, and PSEN1 CDSs select slightly higher FDs compared to the mRNA, while PSEN2 CDSs FDs are lower. The correlation coefficient for these sequences is 0.969. A comparative study of differentially expressed MAPK signaling pathway lncRNAs in pancreatic cancer cells shows a correlation of 0.771. Selection of higher FD CDSs could indicate interaction of Alzheimer's gene products APP, APOE, and PSEN1. Including hypocretin sequences (where all CDSs have higher fractal dimensions than mRNA) in the APP, APOE, and PSEN1 sequence analyses improves correlation, but the inclusion of erythropoietin (where all CDSs have higher FD than mRNA) would suppress correlation, suggesting that HCRT, a hypothalamus neurotransmitter related to the wake/sleep cycle, might be better when compared to EPO, a glycoprotein hormone, for targeting Alzheimer's disease drug development. Fractal dimension and entropy correlation have provided supporting evidence, consistent with evolutionary studies, for using a zebrafish model together with a mouse model, in HCRT drug development.

## 1. Introduction

The instructions of a genetic sequence are carried by the fluctuations or variations in the nucleotide bases along the sequence. The bioinformatics of a sequence can be studied if the sequence is modeled as a series based on the nucleotide atomic number of the nucleotides A, T, C, and G. A recent study on such fluctuation in the FOXP2 gene pathway has been reported [1]. The fractal dimension and the Shannon entropy were found to have a negative correlation (*R* ^2^ = 0.85  *N* = 12) for the FOXP2 regulated “accelerated conserved-non-coding” sequences in human fetal brain. In general, fractal dimension and the Shannon entropy generate a 2D map representing a set of genetic sequences. For example, using the human Y chromosome, which contains 429 genetic sequences according to the http://ncbi.nlm.nih.gov/mapview/ database, the listed sequences have fractal dimension values from 1.92 to 2.06 and the Shannon dinucleotide entropy from 3.0 to 3.8 bits per base. Fractal dimension, being a nucleotide position sensitive measure, would be related to the richness in the embedded informatics associated with the sequence. In terms of transcription and translation, fractal dimension may be related to a docking energy parameter with similarity to the concept of roughness in a zipper and assembly traffic analogy for the docking interactions. In addition to CDS, mRNA sequences are often embedded with intronic regions. Noncoding RNA sequence with more than 200 base pair in length has been used to label a long non-coding RNA (lncRNA) regardless of intronic or intergenic in origin. This project uses fractal dimension of mRNA and coding DNA sequences (CDS) to probe the lncRNAs within the mRNAs (but not the CDS) in Alzheimer's disease genes, namely, APP or AD1, APOE or AD2, PSEN1 or AD3, and PSEN2 or AD4. The lncRNA sequences have been shown to be involved in significant regulatory functions. The lncRNA SPRY4-IT1 sequence was reported to have migrated to the cytoplasm, and upregulation was observed in melanoma cells [2]. Differential expressions in lncRNA upon radiation of HeLa and MCF-7 cells and in glioblastoma pathogenesis have been reported [3, 4]. The lncRNA relationship to cellular genetic product stability has been reported in the mouse model [5]. Microarray data analysis linked lncRNA to Huntington's disease [6], and the lncRNA role in neurological disorders and cancers has been reviewed [7, 8]. The lncRNAs becoming a new cancer diagnostic and therapeutic gold mine have been postulated [9]. Despite all these activities, only a few computation analysis results on the lncRNAs have been reported to our knowledge. A comparative study of lncRNAs with 3′ untranslated regions (3′ UTRs) in protein coding RNA sequences has revealed parallel structure in the studied sequences, consistent with the presence of similar evolutionary constraints [10].

This project focuses on the study of disease related genetic sequences. The lncRNAs within the mRNA sequences in Alzheimer's disease genes, namely, APP or AD1, APOE or AD2, PSEN1 or AD3, and PSEN2 or AD4, have been analyzed in terms of fractal dimension computation and correlation analysis. The exploratory hypothesis that the lncRNA sequences embedded in a transcribed mRNA sequence would exhibit correlation in Alzheimer's disease genes has been studied in a comparative fractal dimension model of mRNA sequences versus coding DNA sequences (CDSs), which do not include the lncRNA sequences. 

## 2. Materials and Methods

The data used in this study was downloaded from GenBank according to the following Gen-ID numbers. The studied human genes are APP-Gen-ID-351 containing 10 mRNA variants, APOE-Gen-ID-348, PSEN1-Gen-ID-5663 having 2 mRNA variants, PSEN2-Gen-ID-5664 having two mRNA variants, HCRT-Gen-ID-3060, HCRTR1-Gen-ID-3061 (HCRT Receptor-1), HCRTR2-Gen-ID-3062 (HCRT Receptor-2), EPO-Gen-ID-2056, and EPOR-Gen-ID-2057. The MAPK signaling pathway gene accession numbers have been listed in the report of differential expression of long non-coding intronic RNAs in pancreatic cancer cells [11]. The Allen Brain Atlas database has been accessed at http://brain-map.org.

A sequence with a relatively low nucleotide variety would have low Shannon's entropy (more constraints) in terms of the set of 16 possible dinucleotide pairs. A sequence's entropy can be computed as the sum of (*p*_*i*_)∗log⁡(*p*_*i*_) over all states *i*, and the probability *p*_*i*_ can be obtained from the empirical histogram of the 16 di-nucleotide pairs. The maximum entropy is 4 binary bits per pair for 16 possibilities (2^4^). For mono-nucleotide consideration, the maximum entropy is two bits per mononucleotide with four possibilities (2^2^). In general, the monoentropy is proportional to di-nucleotide entropy with *R* ^2^ > 0.75 for the reported sequences in the paper.

 Roughly speaking, fractal dimension measures the complexity of a self-similar sequence. For a 1D sequence such as a DNA sequence, a fractal dimension near 2 indicates great complexity, while one closer to 1 would indicate little complexity, variety, or information. Among the various fractal dimension methods, the Higuchi fractal method is well suited for studying signal fluctuation [12]. A random spatial series with equal spatial steps can be modeled as a brightness signal in time such that the time series analysis tools can be used for spatial series analysis. The spatial intensity (Int) random series with equal intervals could be used to generate a difference series (Int(*j*) − Int(*i*)) for different lags in the spatial variable. The nonnormalized apparent length of the spatial series curve is simply *L*(*k*) = Σ|(Int(*j*) − Int(*i*))| where the sum is for all pairs where *j*-*i* = *k*. The number of terms in a *k*-series varies, and normalization must be used to get the series length. If the Int(*i*) is a fractal function, then the log⁡(*L*(*k*)) versus log⁡(1/*k*) should be a straight line with the slope equal to the fractal dimension. Higuchi incorporated a calibration division step such that the maximum theoretical value is calibrated to the topological value of 2. The details of the calculation method are given in the literature [12]. Numerical examples of the fractal dimension computation can be found in our earlier reports [13, 14]. For clarity, a Matlab implementation of the algorithm we used is listed below. “data” is an array loaded with the input sequences (one in each column). “width” is the number of sequences loaded in the data array. “max⁡⁡*K* ” is cutoff for the maximum distance between *j*-*i* pairs. A max⁡ *K* of 7 was used for this paper, although other values for max⁡⁡*K* gave very similar results.

Consider

 % calculate Length vectors for each column
 
*L* = zeros(max⁡⁡*K*, width);
 for *k* = 1 : max⁡⁡*K*,
  data2 = circshift(data, *k*);
  data2 = abs(data2 − data);
  data2 (1 : *k*, :) = 0; % remove end effects
  
*L*(*k*, :) = sum(data2)/*k*/*k*∗(height − 1)/(height − *k*);
  end

 % calculate slopes (FDs)
 slope = zeros(1, width);
 for *i* = 1 : width,
  temp = 1 : 1 : max⁡⁡*K*;
  log⁡⁡*k* = log⁡⁡(1./temp)′;
  
*X* = [ones(size(log⁡⁡*k*))log⁡⁡*k*];
  
*Y* = log⁡⁡(*L*(:, *i*));
  
*a* = *X*∖*Y*;
  FD(*i*) = *a*(2);
 End

## 3. Results of Fractal Analysis

The ratio of CDS length to mRNA length ranges from 0.23 to 0.78 in the studied Alzheimer's disease sequences. A negative correlation with *R* ^2^ of 0.61 was found for fractal dimension and the ratio of CDS length to mRNA length, using the 10 variant sequences in APP mRNA. The increase of fractal dimension with decreasing ratio could be related to some systematic properties in mRNA variant formation in the APP gene. It would appear that increasing the lncRNA length portion relative to the CDS length portion would correlate with increasing fractal dimension in the APP mRNA variants.

**Figure 1:**
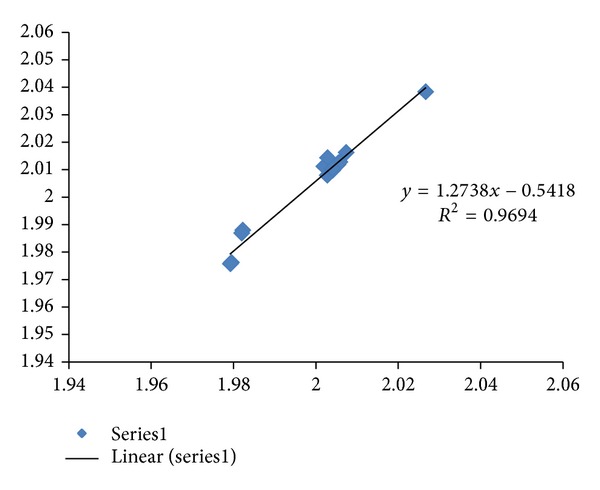
The fractal dimension correlation of the APP, APOE, PSEN1, and PSEN2 CDSs versus mRNAs in Alzheimer's disease is displayed with *R* ^2^ of 0.969 (*N* = 15 Series1). The *y*-axis represents the fractal dimension (FD) of the CDSs, and the *x*-axis represents the FD of the mRNAs. Deletion of PSEN2 Variant 1 (1.9793, 1.9748) and Variant 2 (1.9791, 1.9743) where CDSs have lower fractal dimension values as compared to the mRNA sequences, which would give *R* ^2^ of 0.979, *N* = 13.

**Figure 2:**
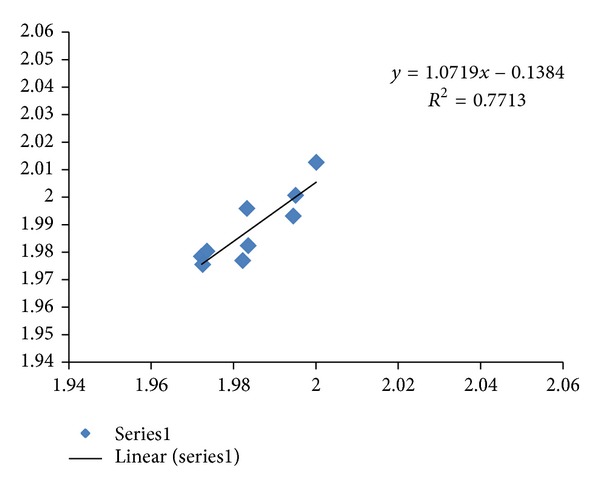
The fractal dimension correlation of the differentially expressed MAPK signaling pathway long noncoding intronic RNA in pancreatic cancer cells with a correlation of *R* ^2^ = 0.771  (*N* = 9 Series1). The *y*-axis represents the fractal dimension (FD) of the CDSs, and the *x*-axis represents the FD of the mRNAs. Deletion of MAP3K1 (1.9945, 1.9929), MAP3K14 (1.9822, 1.9768), and RAPGF2 (1.9835, 1.982) where CDSs have lower fractal dimension as compared to the mRNA sequences which would give *R* ^2^ of 0.950 (*N* = 6). The sequence GenBank accession numbers have been listed in [11].

The fractal dimension correlation of the CDSs versus mRNAs in Alzheimer's disease is displayed in Figure 1 with *R* ^2^  of 0.969 (*N* = 15). All of the studied Alzheimer's disease sequences show higher fractal dimensions in the CDSs as compared to the mRNAs, except for the PSEN2 Variant 1 and Variant 2. Similarly, a comparative study of the differentially expressed MAPK signaling pathway of long non-coding intronic RNA in pancreatic cancer cells is displayed in Figure 2 with a correlation of *R* ^2^ = 0.771 [11]. The MAPK signaling pathway has been reported to involve 9 mRNAs in differential expression of long non-coding intronic RNA in [11]. They are ARRB1, ATF2, MAPK1, MAP2 K5, MAP3 K1, MAP3 K14, PPP3CB, RAPGF2, and TGFbR2. The CDSs of MAP3 K1, MAP3 K14, and RAPGF2 have lower fractal dimension values as compared to the mRNAs. 

**Figure 3:**
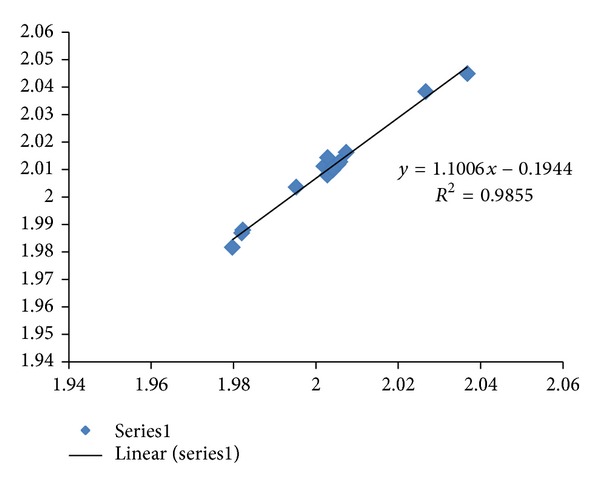
The fractal dimension correlation of the APP, APOE, PSEN1, HCRT, HCRT-R1, and HCRT-R2 CDSs versus mRNAs in Alzheimer's disease is displayed with *R* ^2^ of 0.985 (*N* = 16 Series1). The *y*-axis represents the fractal dimension (FD) of the CDSs, and the *x*-axis represents the FD of the mRNAs.

The systematic selection of higher fractal dimension CDSs could be indicative of certain characteristic interaction of the Alzheimer's gene products APP, APOE, and PSEN1 where a correlation with *R* ^2^ of 0.979 (*N* = 13) was obtained. Hypocretin (orexin) loss in Alzheimer's disease patients has been reported [15]. A brain scan study on a group of young adults has revealed 1/3 of them are PSEN1 E280A mutation carriers, an accepted hallmark for Alzheimer's disease [16]. The inclusion of hypocretin sequences (where all CDSs have higher fractal dimension values than mRNAs in HCRT, HCRT-R1, and HCRT-R2) in the APP, APOE, and PSEN1 sequence analysis would improve the correlation (*R* ^2^ = 0.985, *N* = 16) as shown in Figure 3. Erythropoietin EPO has been shown to have interaction with dopamine pathways [17–19] and offer protection for neuronal injury [20, 21]. The inclusion of erythropoietin (where all CDSs have higher dimension than mRNA in EPO and EPOR) in the correlation of APP, APOE, and PSEN1 would suppress the correlation (*R* ^2^ = 0.953, *N* = 15). 

## 4. Discussion

The regression intercepts in Figures 1, 2, and 3 are negative, while the slopes are all greater than 1. This indicates selection pressure driving up CDS fractal dimension or a selection pressure against high FD in lncRNA. Whether the negative intercept value would suggest a minimum fractal dimension threshold in mRNA for containing a functional CDS in the studied set of Alzheimer's disease genes needs further investigation. The exploratory hypothesis that the lncRNA sequences embedded in the transcribed mRNAs would exhibit correlation in Alzheimer's disease genes receives supporting evidence in a comparative fractal dimension model of mRNA sequences versus coding DNA sequences (CDSs).

**Figure 4:**
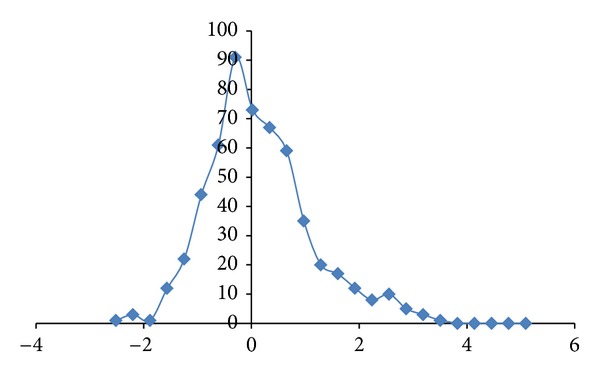
HCRT Receptor-2 expression level distribution in the brain regions (168 regions for each patient) using the 4-patient data from Allen Brain Atlas. The expression level *z*-score values are displayed in the *x*-axis. The displayed line is used as a visual guide. The Skewness of the distribution has been computed to be = 0.91.

**Figure 5:**
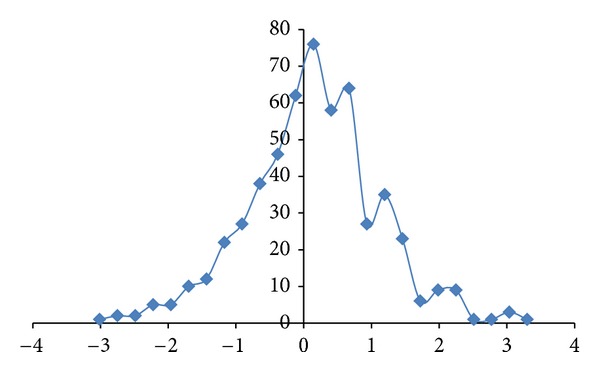
EPO Receptor expression level distribution in the brain regions (168 regions for each patient) using the 4-patient data from Allen Brain Atlas. The expression level *z*-score values are displayed in the *x*-axis. The displayed line is used as a visual guide. The Skewness of the distribution has been computed to be = 0.04.

**Figure 6:**
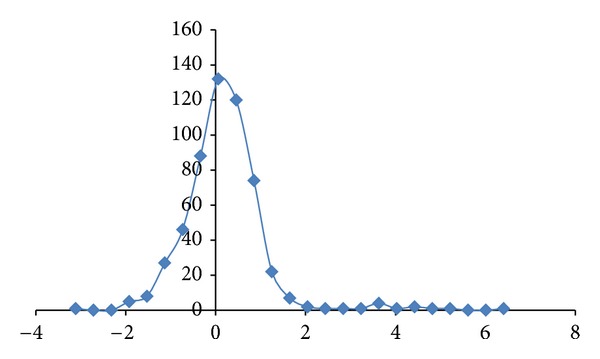
HCRT expression level distribution in the brain regions (168 regions for each patient) using the 4-patient data from Allen Brain Atlas. The expression level *z*-score values are displayed in the *x*-axis. The displayed line is used as a visual guide. The Skewness of the distribution has been computed to be = 2.2.

**Figure 7:**
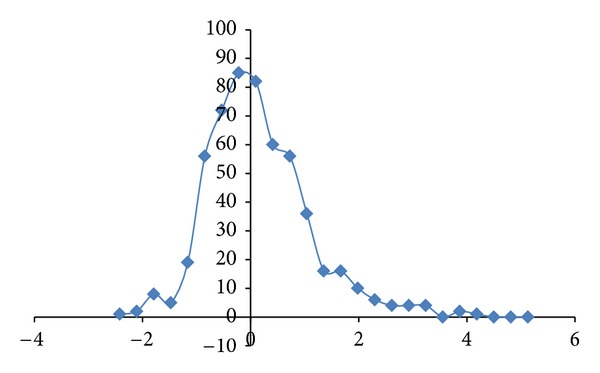
EPO expression level distribution in the brain regions (168 regions for each patient) using the 4-patient data from Allen Brain Atlas. The expression level *z*-score values are displayed in the *x*-axis. The displayed line is used as a visual guide. The Skewness of the distribution has been computed to be = 1.1.

The correlation results suggest a hypothesis where HCRT, a neurotransmitter only produced in the hypothalamus and related to the wake/sleep cycle, could be a relatively more important candidate as a blocker or promoter when compared to EPO, a glycoprotein hormone produced by kidney and liver, for targeting drug development with application to Alzheimer's disease clinical trials. The HCRT hypothesis would be consistent with MRI brain scans (168 regions) containing microarray array expression level data from the Allen Brain Atlas database. The brain scan data analysis has showed higher Skewness value in HCRT Receptor-2 expression level distribution (Figure 4) in the brain as compared to EPO Receptor distribution (Figure 5). The reasoning follows the fact that hypocretin Receptor expression level distribution would have a positive long tail representing high expression level and high demand for HCRT in the brain as compared to EPO receptor expression level distribution. The Skewness value would be 0.91 for HCRT Receptor-2 (Figure 4) versus 0.04 for EPO Receptor (Figure 5), a factor difference of about 20. The HCRT and EPO expression level distributions in the brain are displayed in Figures 6 and 7, respectively. The Skewness value would be 2.2 for HCRT (Figure 6) versus 1.1 for EPO (Figure 7), a factor difference of about 2. The large factor difference in receptor expression level in the brain would influence the selection of targeted receptors in drug development. 

**Figure 8:**
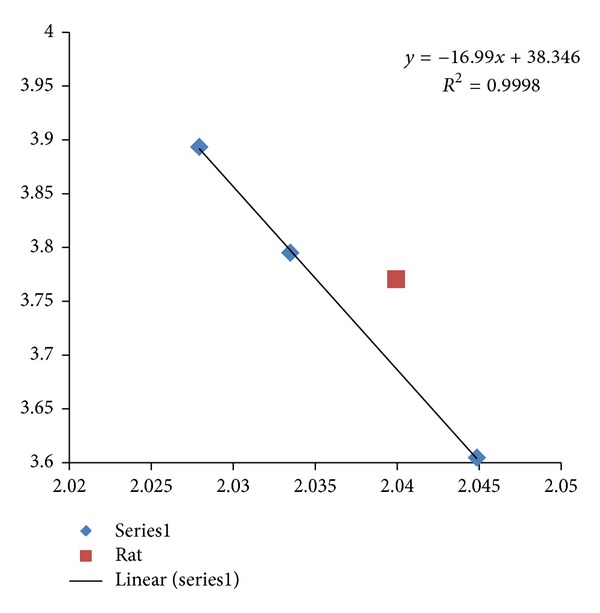
A plot of fractal dimension versus entropy for HCRT CDSs in human, mouse, and zebrafish is displayed with *R* ^2^ of 0.9998 and an adjusted *R* ^2^ of 0.9996 (Series1 diamonds). The fractal dimension is represented on the *x*-axis, and the dinucleotide entropy is represented on the *y*-axis. Rat HCRT CDS would be viewed as an outlier (square) from the regression analysis of human (highest fractal dimension), mouse and zebrafish (lowest fractal dimension). GenBank information: mouse Mus musculus HCRT has Gen-ID-15171, rat Rattus norvegicus HCRT has Gen-ID-25723, and zebrafish Danio rerio HCRT has Gen-ID-613239.

**Figure 9:**
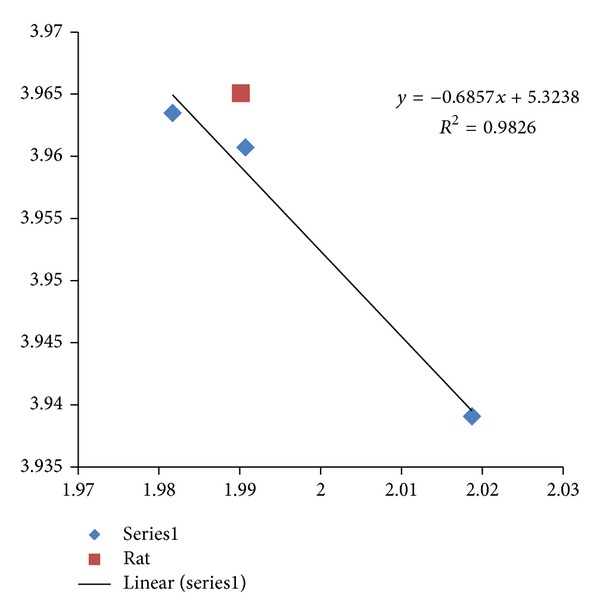
A plot of fractal dimension versus entropy for HCRT-R2 CDSs in human, mouse, and zebrafish is displayed with *R* ^2^ of 0.982 and an adjusted *R* ^2^ of 0.965 (Series1 diamonds). The fractal dimension is represented on the *x*-axis, and the dinucleotide entropy is represented on the *y*-axis. Rat HCRT-R2 CDS would be viewed as an outlier (square) from the regression analysis of human (lowest fractal dimension), mouse and zebrafish (highest fractal dimension). GenBank information: mouse Mus musculus HCRT has Gen-ID-387285, rat Rattus norvegicus HCRT has Gen-ID-25605, and zebrafish Danio rerio HCRT has Gen-ID-561260.

Mouse model has become a popular choice in drug development since evolution has been a corner stone for the understanding of biology. A plot of fractal dimension versus entropy for HCRT CDSs in human, mouse, and zebrafish is displayed in Figure 8 with an adjusted *R* ^2^ of 0.9996, and rat HCRT CDS would be viewed as an outlier from the regression analysis of human, mouse, and zebrafish. The regression result would be consistent with an evolutionary trend where the human HCRT has the highest fractal dimension, and zebrafish HCRT has the lowest fractal dimension. Similar fractal dimension entropy plot on HCRT-R2 is displayed in Figure 9 with an adjusted *R* ^2^ of 0.965, and rat HCRT-R2 CDS would be viewed as an outlier. The regression result would be consistent with an evolutionary trend, where the human HCRT-R2 has the lowest fractal dimension, and zebrafish HCRT-R2 has the highest fractal dimension. The high fractal dimension human HCRT combination of low fractal dimension human receptor HCRT-R2 would be consistent with a docking complimentary relationship as discussed above in terms of the significance of fractal dimension as an associated parameter for roughness matching in a zipper analogy for the understanding of transcription and translation. The folding of lncRNA could be important for further studies of docking and regulation in terms of sequence fractal dimension computation, and metal controlled folding RNA could serve as a starting platform with UV Circular Dichroism and Synchrotron based X-ray absorption spectroscopy structural data [22]. In any event, when a fractal dimension entropy map is used as a tool for evolutionary pressure study beyond simple classification, a strong correlation would lend quantitative support to the choice of mouse model and zebrafish model in HCRT drug development. Extension of fractal dimension computation and correlation analysis to the recently published dataset of expressed lncRNAs in zebrafish embryogenesis would help HCRT drug development [23].

**Figure 10:**
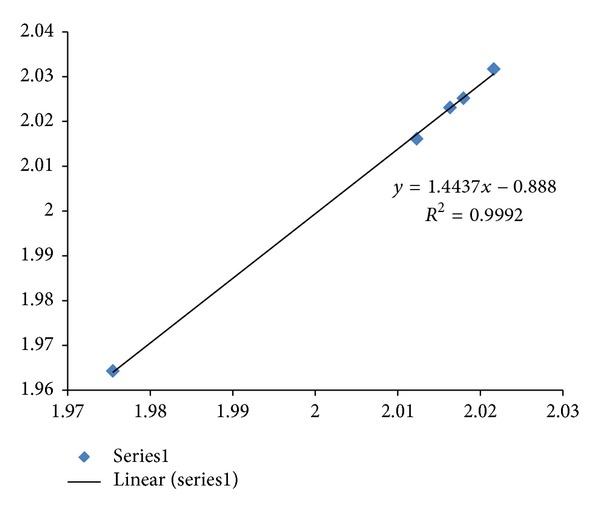
The fractal dimension correlation of the TYROBP-Variants 1–4 and TREM2 CDSs versus mRNAs involved in Late-onset Alzheimer's disease. *R* ^2^ = 0.9992 (*N* = 5). Inclusion of APOE (2.0267, 2.0382) would appear at the uppermost right corner in contrast to TREM2 at the lowest left corner and would give *R* ^2^ = 0.9993, *N* = 6 (adjusted *R* ^2^ = 0.99915, *N* = 6). For comparison, similar correlation analysis on the mouse and Bos taurus TYROBP, TREM2, and APOE would give *R* ^2^ values of 0.89 (*N* = 3), and 0.45 (*N* = 3) respectively. GenBank information: human TYROBP has Gen-ID-7305, human TREM2 has Gen-ID-54209, mouse TYROP has Gen-ID-22177, mouse TREM2 has Gen-ID-83433, mouse APOE has Gen-ID-11816, Bos taurus TYROP has Gen-ID-282390, Bos taurus TREM2 has Gen-ID-506467, and Bos taurus APOE has Gen-ID-281004.

**Figure 11:**
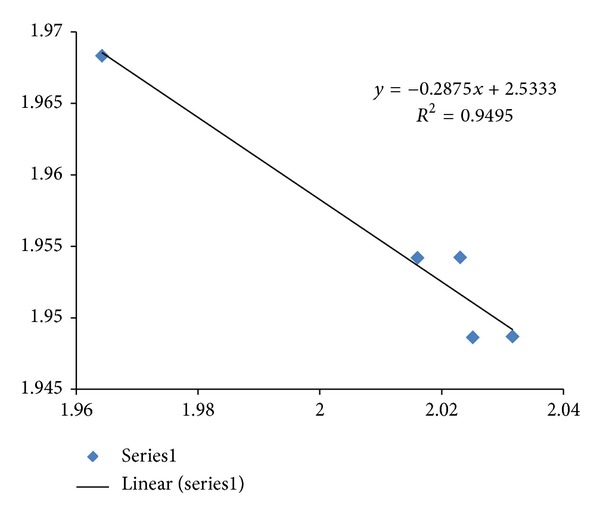
Fractal dimension versus entropy for TYROBP-Variant 1, TYROBP-Variant 2, TYROBP-Variant 3, TYROBP-Variant 4, and TREM2 CDSs involved in Late-onset Alzheimer's disease in human. The fractal dimension is represented on the *x*-axis, and the mononucleotide entropy is represented on the *y*-axis. TYROBP-Variant 1 has the highest fractal dimension and TYROBP-Variant 2 has the second lowest fractal dimension, and TREM2 has the lowest fractal dimension. Inclusion of APOE (2.0382, 1.8673) would suppress *R* ^2^ to 0.32.

Recently the R47H variant of TREM2 was reported to be associated with Late-onset Alzheimer's disease (LOAD) [24, 25]. The human TREM2 is known as an innate immune receptor and signals through TYROBP (agonist with 4 mRNA variants and 4 CDS variants) to clear the damaged tissue and reduce inflammation. The fractal dimension computation and correlation is displayed in Figure 10 with *R* ^2^ = 0.999, *N* = 5. The BLASTN comparison of TREM2 (lowest left corner data point in Figure 10) shows *E* = 0.11, given a CDS of 693-nucleotide sequence versus a 366-nucleotide sequence within the mRNA obtained by adding the beginning and ending non-coding regions together. Similar BLASTN comparison of TYROBP Variant 1 (uppermost right corner data point in Figure 10) had returned a null result, given a CDS of 342-nucleotide sequence versus a 266-nucleotide sequence within the mRNA obtained as described above. The fact that the CDS and mRNA of the studied sequences have similar fractal dimension values but show little or no relationship under BLAST investigation would suggest a selection process, and the correlation showing *R* ^2^ of 0.9992 (*N* = 5) among the 4 variants and the receptor would suggest a systematic selection process, consistent with the CDSs entropy versus fractal dimension plot having *R* ^2^ of 0.949 (*N* = 5) in Figure 11. 

As [24] pointed out, the apolipoprotein E (APOE) malfunction still remains as the most important sequence variant that would be risk of Late-onset Alzheimer's disease. The inclusion of APOE in Figure 10 would give *R* ^2^ of 0.9993 (*N* = 6), suggesting a very stringent regulation in selecting CDSs from mRNAs in the studied LOAD sequences. For comparison, similar correlation analysis on the mouse and Bos taurus TYROBP, TREM2, and APOE would give *R* ^2^ values of 0.89 (*N* = 3) and 0.45 (*N* = 3), respectively. The BLASTN comparison of human APOE had returned *E* = 0.11, given a CDS of 954-nucleotide sequence versus a 269-nucleotide sequence within the mRNA obtained by adding the beginning and ending non-coding regions together. The inclusion of HCRT and EPO informatics would suppress the correlation to *R* ^2^ values of 0.973 (*N* = 9) and 0.927 (*N* = 8), respectively, suggesting that HCRT drugs could be a better choice for treating Late-onset Alzheimer's disease as compared to EPO drugs. The inclusion of APOE would give *R* ^2^ of 0.32 in the entropy versus fractal dimension graph in Figure 11. The APOE sequence has the lowest entropy among the studied sequences, and all CDSs have lower entropy than mRNAs in the Late-onset Alzheimer's disease studied sequences. The APOE mononucleotide entropy of 1.8673 would suppress very slightly the mono-nucleotide correlation of mRNA versus CDS in Late-onset Alzheimer's disease studied sequences from *R* ^2^ of 0.9948 (*N* = 5) to 0.9944 (*N* = 6). The Late-onset Alzheimer's disease studied mRNAs and CDSs show very high correlation in fractal dimension (*R* ^2^ of 0.999) and entropy (*R* ^2^ of 0.994), consistent with a Late-onset Alzheimer's disease lncRNA hypothesis of high fractal dimension satisfied by low entropy in CDSs selection by deleting the lncRNAs with low entropy values about 1.91 except for TREM2 lncRNA having 1.995 bits per nucleotide type.

High correlation results are also observed in two other neurodegenerative disease involving TYROBP. The Nasu-Hakola disease, a disorder affecting both brain and bone, is known to be related to the malfunctioning of TREM2 or TYROBP [26]. The CSF1R (a microglial receptor) where malfunctioning is associated with a corticobasal syndrome called hereditary diffuse leukoencephalopathy with spheroids was reported to be cosignaling with TYROBP [27]. The addition of CSF1R in Figure 10 would reduce the correlation from 0.999 (*N* = 5) to 0.992 (*N* = 6). The exploratory study of noncoding RNA by comparing mRNA versus CDS informatics has revealed regularity. An examination of Figure 1 with *R* ^2^ 0.969 (*N* = 15) and Figure 10 with *R* ^2^ 0.999 (*N* = 6 including APOE) would suggest that the noncoding RNA assembly process in Late-onset Alzheimer's disease would involve relatively highly systematic process or processes as compared to familial early-onset Alzheimer's disease with APP, PSEN1, PSEN2, and APOE. The project has used *R* ^2^ differences in the order of 0.02 to be the demarcation in the lncRNA investigation by comparing mRNAs versus CDSs in a cluster of disease related sequences. The mRNA versus CDS informatics comparative method could be a supplement to the well-accepted BLAST method. Further investigations using fractal analysis for neurodegenerative disease sequences and currently targeted receptors would be productive. 

## 5. Conclusions

 The long noncoding RNAs (lncRNAs) within the mRNA sequences in Alzheimer's disease genes, namely, APP, APOE, PSEN1, and PSEN2, have been analyzed in terms of fractal dimension computation and correlation analysis. The results show that APP, APOE, and PSEN1 CDSs select slightly higher fractal dimensions as compared to the mRNA sequences with a pattern evidenced by correlation coefficient of *R* ^2^ = 0.979  (*N* = 13 including variants). Inclusion of the 2 variants in PSEN2 where CDSs have lower fractal dimension values than mRNAs would yield *R* ^2^ of 0.969 (*N* = 15). The systematic selection of higher fractal dimension CDSs could be indicative of characteristic interaction of Alzheimer's gene products APP, APOE, and PSEN1. The inclusion of hypocretin sequences would improve the correlation (*R* ^2^ = 0.985, *N* = 16) but inclusion of erythropoietin would suppress the correlation (*R* ^2^ = 0.953, *N* = 15), suggesting that HCRT could be a relatively more important candidate as a blocker or promoter when compared to EPO for targeting in drug development with application to Alzheimer's disease clinical trials. The HCRT hypothesis would be consistent with MRI brain scan containing microarray expression level data from the Allen Brain Atlas database that shows higher Skewness value in HCRT receptor expression level distribution as compared to EPO receptor expression level distribution in the brain. Study of sequence fractal dimension and entropy correlation has provided quantitative supporting evidence, consistent with evolutionary studies, for using zebrafish model together with mouse model, in HCRT drug development. The TREM2 and TYROBP mRNAs reported recently in Late-onset Alzheimer's disease also yield a correlation of *R* ^2^ of 0.999 (*N* = 6) using similar informatics analysis, but HCRT informatics inclusion would suppress the correlation slightly as compared to the EPO informatics inclusion.
